# Global research trends and hotspots of hyperhidrosis: a bibliometric analysis (2008–2023)

**DOI:** 10.3389/fsurg.2025.1559951

**Published:** 2025-04-22

**Authors:** Tong Zhi, Chengcheng Zhao, Xin Xiang, Ming Yao, Huadong Ni

**Affiliations:** Department of Anesthesiology and Pain Research Center, The Affiliated Hospital of Jiaxing University, Jiaxing, China

**Keywords:** hyperhidrosis, bibliometric, hotspots, VOS viewer, compensatory sweating

## Abstract

**Background:**

Recent studies have demonstrated significant advancements in the treatment of hyperhidrosis. However, a bibliometric analysis of relevant studies in this field is notably lacking. This study aims to provide a detailed analysis of research trends and key areas of interest in hyperhidrosis over the last 16 years using bibliometric methods.

**Methods:**

We searched the Web of Science Core Collection (WoSCC) database for hyperhidrosis-related publications from 2008 to 2023 and conducted bibliometric analysis using VOS viewer and the R package “bibliometrix.”

**Results:**

The main research institutions involved in this study are the University of São Paulo, Hospital Israelita Albert Einstein, Yonsei University and Fujian Medical University, with a total of 728 articles included from 52 countries. Authors from these institutions have published in top journals, with Dermatologic Surgery being the most popular journal and the Journal of the American Academy of Dermatology being the most cited. A total of 2,830 authors have contributed to this field, with prominent researchers including Nelson Wolosker, Paulo Kauffman, Pedro Puech-Leão, Jose Ribas Milanez de Campos, and Dee Anna Glaser. Nelson Wolosker stands out as the most co-cited author. The primary focus of research in this area is on the treatment of hyperhidrosis and the prevention of post-operative complications. Emerging re-search hotspots include keywords such as “botulinum toxin,” “oxybutynin,” “sympathectomy,” “iontophoresis,” and “compensatory sweating”.

**Conclusion:**

The most prevalent academic emphasis within this field remains the treatment of hyperhidrosis and the management of compensatory hyperhidrosis. Despite this academic preponderance, there is a compelling necessity to foster enhanced collaboration and exchange between disparate countries and institutions.

## 1 Introduction

Hyperhidrosis (HH) is a disorder characterized by pathologically focal or generalized excessive sweating, with autonomic dysfunction as one of the possible pathophysiological mechanisms (1, 2). It is classified into primary hyperhidrosis and secondary hyperhidrosis. Primary Hyperhidrosis (PHH) affects approximately 2% of the population and usually starts in adolescence with localized sweating from the armpits, hands, face, or feet (3). On the other hand, Secondary Hyperhidrosis (SHH) typically manifests as generalized sweating and is often linked to underlying health conditions or substance abuse. The following factors are associated with drug-induced hyperhidrosis: antipyretic and analgesic drugs, hypoglycemic drugs, antidepressants, etc. Additional factors include tumor tissue affecting the thermoregulatory center and obesity resulting in increased thermogenesis, which may collectively lead to secondary hyperhidrosis. Primary Hyperhidrosis is known to be a stigmatizing condition that inflicts a significant mental and physical toll on afflicted individuals. Patients often grapple with feelings of embarrassment and apprehension about their symptoms and the reactions of others. This, in turn, can profoundly impact their self-esteem, social interactions, intimate relationships, and professional endeavors. The Health-Related Quality of Life (HRQoL) of HH patients is typically lower compared to those without the condition or individuals with other chronic skin ailments (4, 5). Moreover, individuals with HH face an increased risk of developing depression, anxiety, and skin infections. These additional health challenges can further exacerbate the overall disease burden experienced by HH patients, underscoring the multifaceted impact of this condition on both physical and psychological well-being (6, 7).

Bibliometrics is a method of analyzing literature that examines the output and status of publications in a specific field of study from both numerical and qualitative perspectives. During the analysis process, information about authors, keywords, journals, countries, institutions, references, and other relevant aspects of the field can be gathered. Commonly used bibliometric tools, such as VOS viewer (8) and the R package “bibliometrix”, help visualize the results of these analyses. These tools have been widely used in medical fields, including oncology (9), orthopedics (10), and rheumatology (11). However, despite their broad application, no bibliometric studies on hyperhidrosis have been identified. Therefore, the purpose of this study is to conduct a bibliometric analysis of articles on hyperhidrosis published from 2008 to 2023. Pursuant to the findings of the bibliometric analysis, the field of hyperhidrosis stands poised to embark on a range of potential courses of action. In regard to research direction, it is possible to identify current hotspots. In clinical practice, it is possible to optimize protocols based on the data on the efficacy of different treatments, and to pay attention to the use of emerging technologies in treatment. In medical education, it is necessary to adjust the teaching content to cover cutting-edge knowledge and to guide students to conduct scientific research based on the results of analysis. For clinicians, the analysis of data can facilitate the comprehension of the efficacy and safety of various treatment modalities, thereby enabling the optimization of treatment plans. For researchers, the data can facilitate the identification of research hotspots, such as the study of pathogenesis, and the comprehension of research trends. Furthermore, the identification of the core research team can provide a foundation for cooperation and the integration of resources, thereby facilitating in-depth research on hyperhidrosis.

## 2 Methods

### 2.1 Data source

**Figure 1:**
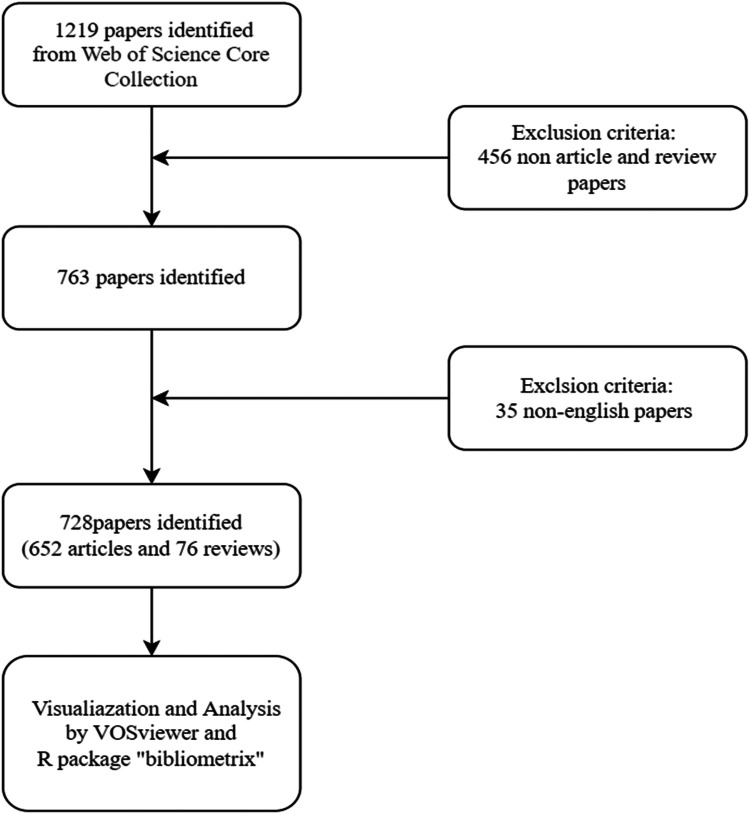
The flowchart for literature screening.

On 31 December 2023, a literature search was conducted using the Web of Science Core Collection (WoSCC) database, specifically the Science Citation Index Expanded (SCIE) index. To ensure the validity and relevance of the retrieved data sources, a title search was chosen over subject and abstract searches, given the potential for retrieving a significant amount of irrelevant literature. In accordance with the search strategy and to ensure the quality of the literature, the inclusion of literature was limited to SCIE publications in English. The search was initiated in 2008, considering the trend of focusing on hyperhidrosis research in the last 15 years. The search query employed in this study on the WoSCC database was formulated as TI = (hyperhidrosis) and LA = (English), with the document types limited to “article” and “review”. The study's literature search strategy and screening process are depicted in Figure 1. To mitigate bias from database updates, data retrieval and export were both performed on December 31, 2023. Since this study did not entail animal subjects or experiments, it was deemed unnecessary to seek ethics committee approval.

### 2.2 Statistical analysis

In our study, we utilized various tools for bibliometric analysis, including VOS viewer (version 1.6.20) and the R package “bibliometrix” (version 4.1.3). VOS viewer is a software capable of extracting key information from a vast number of publications and is commonly employed to establish collaboration, co-citation, and co-occurrence networks (12). In our analysis of publications on hyperhidrosis, we employed a range of analytical tools to gain insights. Firstly, a visual representation was created using VOS viewer. In this representation, each node corresponded to an entity, such as a country, institution, journal, or author. The node size and color indicated the quantity and classification of these entities. The thickness of the connecting lines between nodes indicated the level of collaboration or co-citation among the articles. To investigate the evolution of themes in the literature, we employed the R package “bibliometrix,” which enabled us to construct a global distribution network of hyperhidrosis publications. To provide additional context, data on journal quartile and impact factor were obtained from the 2023 Journal Citation Reports.

## 3 Results

### 3.1 Publication quantitative analysis

**Figure 2:**
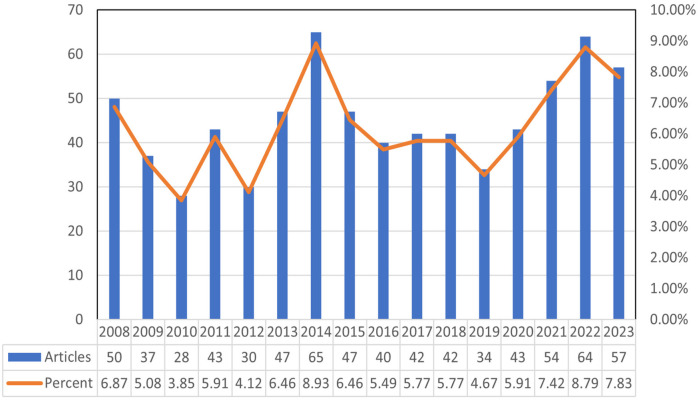
Annual publications from 2008 to 2023.

Our search strategy identified 728 studies on hyperhidrosis in the past 15 years, including 652 articles and 76 reviews. On average, approximately 45.5 papers were published annually. The highest number of papers was in 2014 (*n* = 65), closely followed by 2022 (*n* = 64). Figure 2 shows a small gap in the number of hyperhidrosis related papers published each year.

### 3.2 Co-cited references

**Figure 3:**
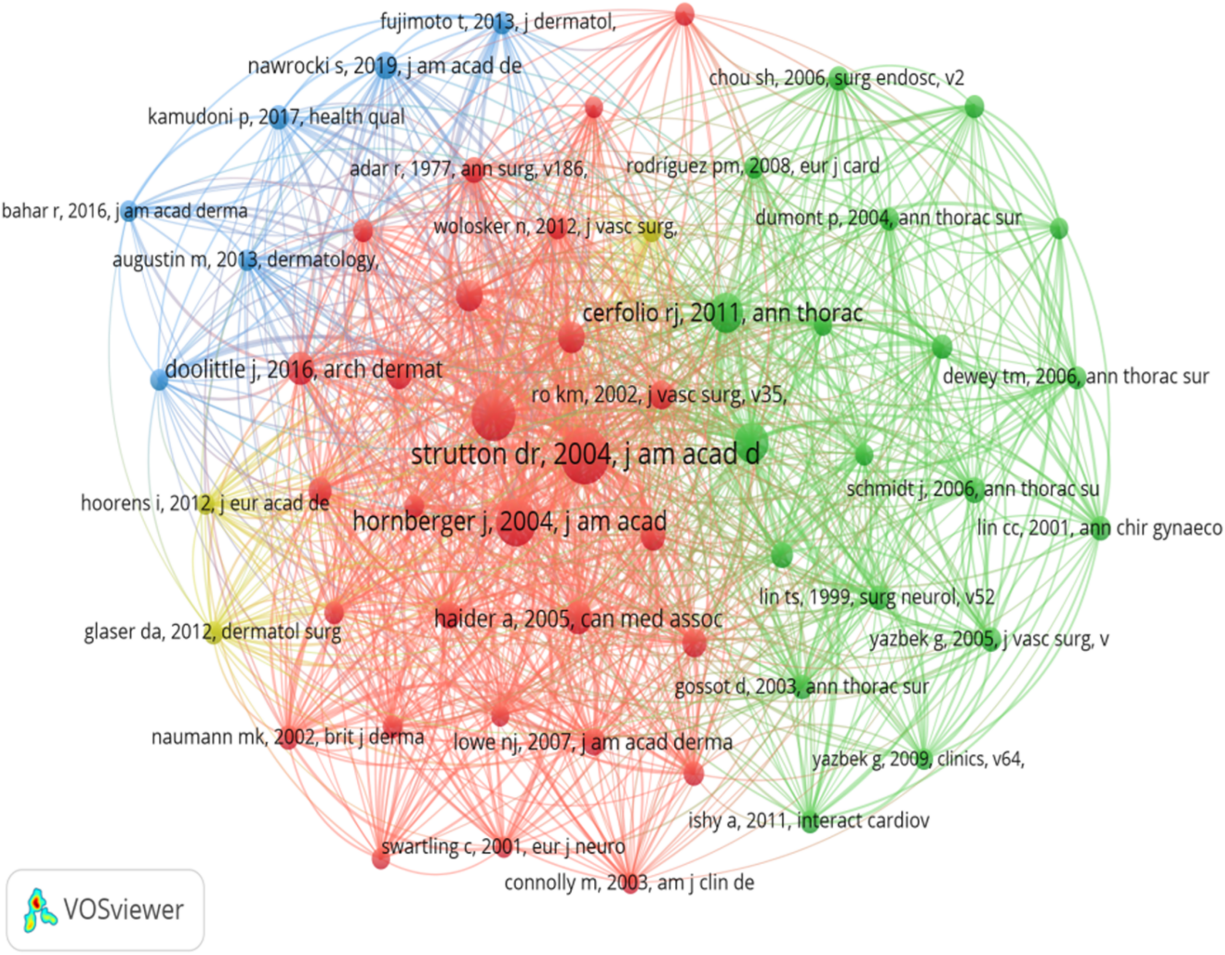
Network map of co-cited references.

Co-citation is defined as the phenomenon in which two or more documents are simultaneously cited by one or more other documents. Over the past 16 years, a total of 6,801 co-cited references pertaining to hyperhidrosis research have been identified. The ten most frequently co-cited references, each with a co-citation count of at least 70 and one reference exceeding 200 citations, are presented in Table 1. It is particularly noteworthy that Strutton DR's 2004 article from the Journal of the American Academy of Dermatology, which focuses on the prevalence of hyperhidrosis in the United States, emerged as the most frequently cited reference. The construction of a co-citation network diagram was pivotal in visualizing the interconnectedness and relationships among these highly co-cited references. references with a cumulative citation count equal to or higher than 35 were chosen. On the one hand, it aligns with the characteristics and data distribution of hyperhidrosis research; 35 citations approximate the threshold at which high-impact literature can be distinguished from general literature, thereby ensuring the representativeness of the included literature. On the other hand, it aligns with the practices in related fields, facilitating the articulation and comparison with other research. Figure 3 illustrates active co-citation relationships, particularly highlighting associations between references such as “Strutton DR, 2004, J Am Acad Dermatol” (13) with “Hornberger J, 2004, J Am Acad Dermatol”, “Solish N, 2007, Dermatol Surg” (14), and “Doolittle J, 2016, Arch Dermatol Res” (15). In the network map of co-cited references, each node signifies a document, with the lines demarcating the co-citation relationships between documents. That is to say, these lines indicate that the documents in question have been co-cited by the same or more other documents. The thickness of the lines connecting the nodes typically corresponds to the strength of the co-references. That is, the presence of a greater number of co-references results in thicker lines.

**Table 1:** The top 10 co-cited references on HH.

Rank	Co-cited reference	Citations
1	Strutton DR, 2004, J Am Acad Dermatol, v51, p241 (13)	249
2	Solish N, 2007, Dermatol Surg, v33, p908 (14)	197
3	Hornberger J, 2004, J Am Acad Dermatol, v51, p274 (1)	171
4	De Campos JRM, 2003, Ann Thorac Surg, v76, p886 (16)	126
5	Cerfolio RJ, 2011, Ann Thorac Surg, v91, p1642 (17)	126
6	Haider A, 2005, Can Med Assoc j, v172, p69 (18)	85
7	Doolittle J, 2016, Arch Dermatol Res, v308, p743 (15)	85
8	Lear W, 2007, Dermatol Surg, v33, ps69 (19)	75
9	Hamm H, 2006, Dermatology, v212, p343 (20)	74
10	Eisenach JH, 2005, Mayo Clin Proc, v80, p657 (21)	71

### 3.3 Contributions of countries and institutions

**Figure 4:**
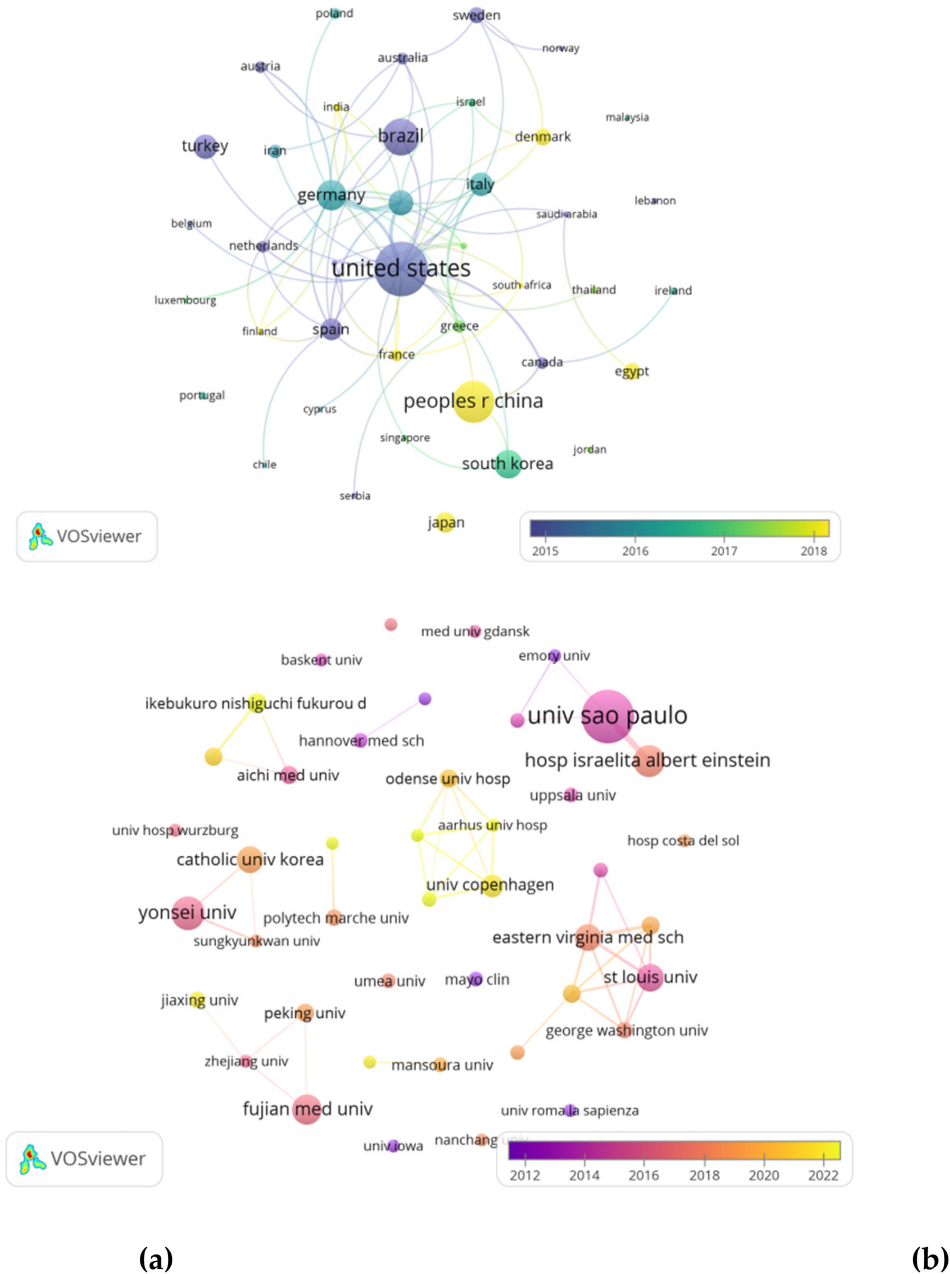
**(a)** Countries collaboration network visualization map. **(b)** Institutional collaboration network visualization map.

The publications included in the study were sourced from 52 countries and 925 institutions, showcasing a global participation in the research field. Notably, the top 10 contributing countries were geographically diverse, representing Asia, North America, South America, and Europe, with a notable concentration in Asia and Europe. The United States emerged as the leading country in terms of publication output, with 154 papers (21.2%), followed by China with 95 papers (13.0%), Brazil with 77 papers (10.6%), and Germany with 55 papers (7.5%). Remarkably, the combined contributions of China and the United States accounted for nearly a third (33.2%) of all publications analyzed. To provide a comprehensive visualization of the data, we selected 41 countries with a publication count of two or more papers to construct a collaborative network diagram (Figure 4a). This collaborative network highlighted the active engagement and relationships between countries based on research collaboration. Notably, close collaborative ties were observed between China and South Korea, as well as between China and Canada. Similarly, the United States demonstrated active collab-oration with Brazil, Germany, South Korea, and the United Kingdom, indicating a robust network of international research partnerships among the countries analyzed.

**Figure 5:**
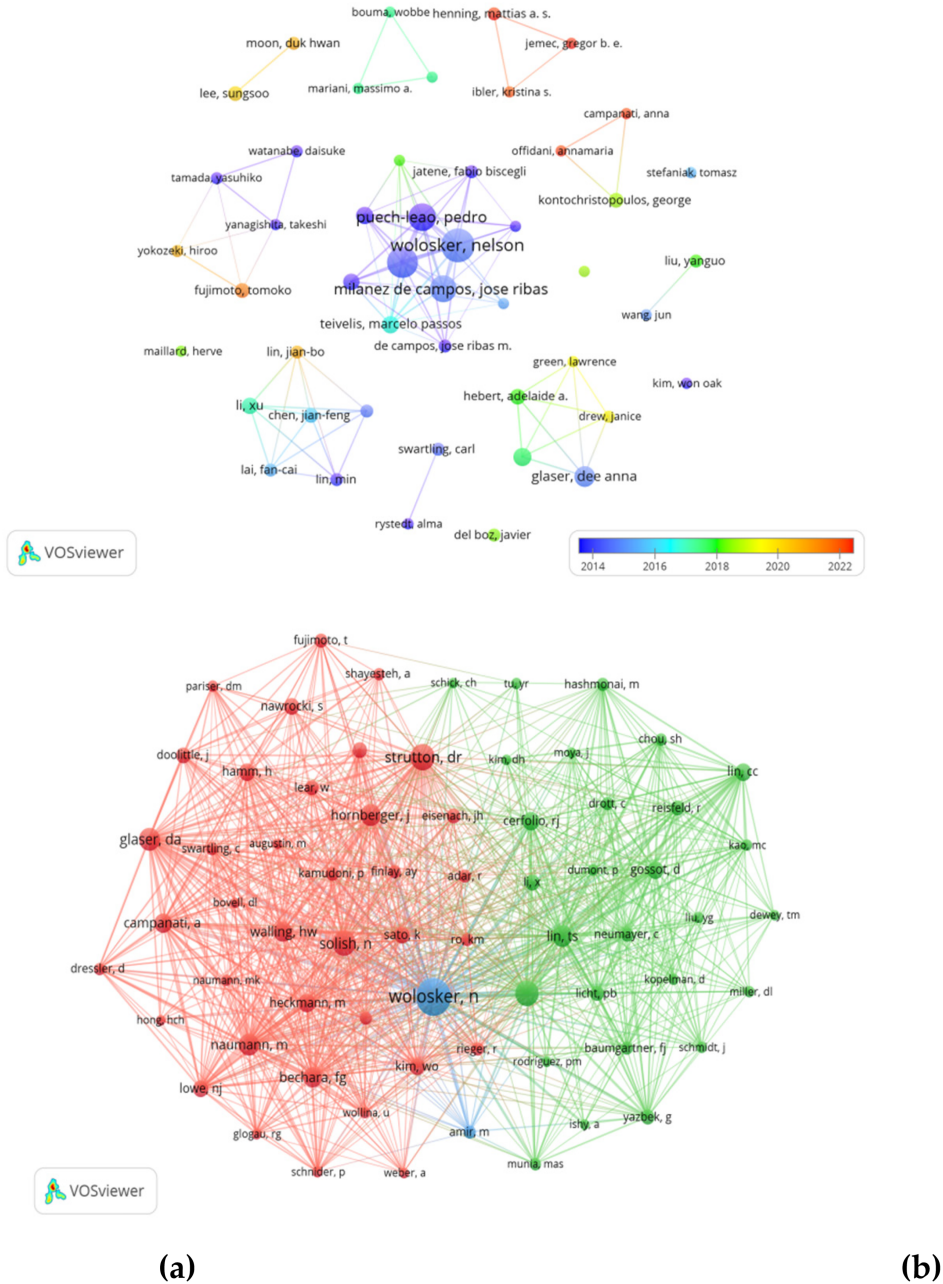
**(a)** Authorship analyses of authors carried out with VOS viewer. **(b)** Co-authorship analyses of authors carried out with VOS viewer.

A total of 925 institutions published papers on hyperhidrosis, with 671 (72.5%) of them contributing only one paper. The top ten institutions, each with a minimum of eight publications, emerged as key players in hyperhidrosis research (Table 2). Leading the pack with 41 articles and a total of 1,124 citations is the University of São Paulo. Following closely were the Albert Einstein Hospital in Israel and Yonsei University, both with 19 publications and more than 250 citations each. To further explore collaborative networks, we focused on 43 institutions that met the criterion of having a minimum of 5 publications. Utilizing this criterion, we constructed a collaborative network based on publication numbers and institutional relationships (Figure 4b). The network analysis revealed a strong collaboration among the University of São Paulo, Albert Einstein Hospital in Israel, and Northwestern University. Moreover, the visual representation in Figure 5 highlighted an active collaboration network involving Fujian Medical University, Zhejiang University, Peking University, and Jiaxing University. The countries/institutional collaboration network visualization map employs a cartographic representation of various types of countries/institutions as nodes and the cooperation relationships between them as connecting lines. The size of the nodes can be adjusted according to specific characteristics or indicators of the countries/institutions. The connecting lines indicate the collaboration relationship between countries/institutions. Different types and intensity of cooperation can be distinguished by the color and thickness of the connecting lines.

**Table 2:** Distribution of the Top 10 countries and institutions on HH.

Rank	Country	Counts	Institution	Counts
1	The United States (North America)	154 (21.2%)	University of São Paulo (Brazil)	41 (5.6%)
2	China (Asia)	95 (13.0%)	Yonsei University (South Korea)	20 (2.7%)
3	Brazil (South America)	77 (10.6%)	Hospital Israelita Albert Einstein (Brazil)	19 (2.6%)
4	Germany (Europe)	55 (7.5%)	Fujian Medical University (China)	17 (2.3%)
5	South Korea (Asia)	49 (6.7%)	Saint Louis University (The United States)	15 (2.1%)
6	The United Kingdom (Europe)	40 (5.5%)	Eastern Virginia Medical School (The United States)	14 (1.9%)
7	Turkey (Asia)	37 (5.1%)	Catholic University of Korea (South Korea)	14 (1.9%)
8	Italy (Europe)	34 (4.7%)	University of Copenhagen (Denmark)	11 (1.5%)
9	Spain (Europe)	30 (4.1%)	Ikebukuro Nishiguchi Fukurou Dermatology Clinic (Japan)	9 (1.2%)
10	Japan (Asia)	27 (3.7%)	Virginia Clinical Research, Inc. (The United States)	8(1.1%)

### 3.4 Authors and co-cited authors

A collaborative network was created based on authors with 4 or more publications (Figure 5a). A total of 2,830 authors participated in hyperhidrosis research, with the top 10 authors publishing more than 10 papers each, as illustrated in Table 3. These authors, namely Nelson Wolosker, Paulo Kauffman, Pedro Puech-Leão, and Jose Ribas Milanez de Campos, were identified as the most influential within the network, due to their significant contributions through the publication of highly relevant papers. The network also revealed close collaborations between several authors. For example, Dee Anna Glaser collaborated closely with David M. Pariser, Adelaide A. Hebert, and Janice Drew. Similarly, Xu Li collaborated with Fancai Lai, Yuanrong Tu, Min Lin, and Jianfeng Chen.

**Table 3:** Distribution of the top 10 authors and co-cited authors on HH.

Rank	Author	Count	Citations	CPP	Co-Cited authors	Citations
1	Nelson Wolosker	44	996	22.6	Nelson Wolosker	503
2	Paulo Kauffman	36	877	24.4	David R Strutton	250
3	Pedro Puech-Leão	30	769	25.6	José Ribas Milanez de Campos	233
4	José RibasMilanez de Campos	28	765	27.3	Nowell Solish	232
5	Dee Anna Glaser	16	331	20.7	Dee Anna Glaser	188
6	David M. Pariser	13	232	17.8	Markus Naumann	176
7	Guilherme Yazbek	12	318	26.5	John Hornberger	171
8	Marcelo Passos Teivelis	12	164	13.7	Torng-Sen Lin	170
9	Xu Li	11	159	14.5	Falk Georges Bechara	146
10	Mariana Krutman	10	243	24.3	Hobart W. Walling	146

**Figure 6:**
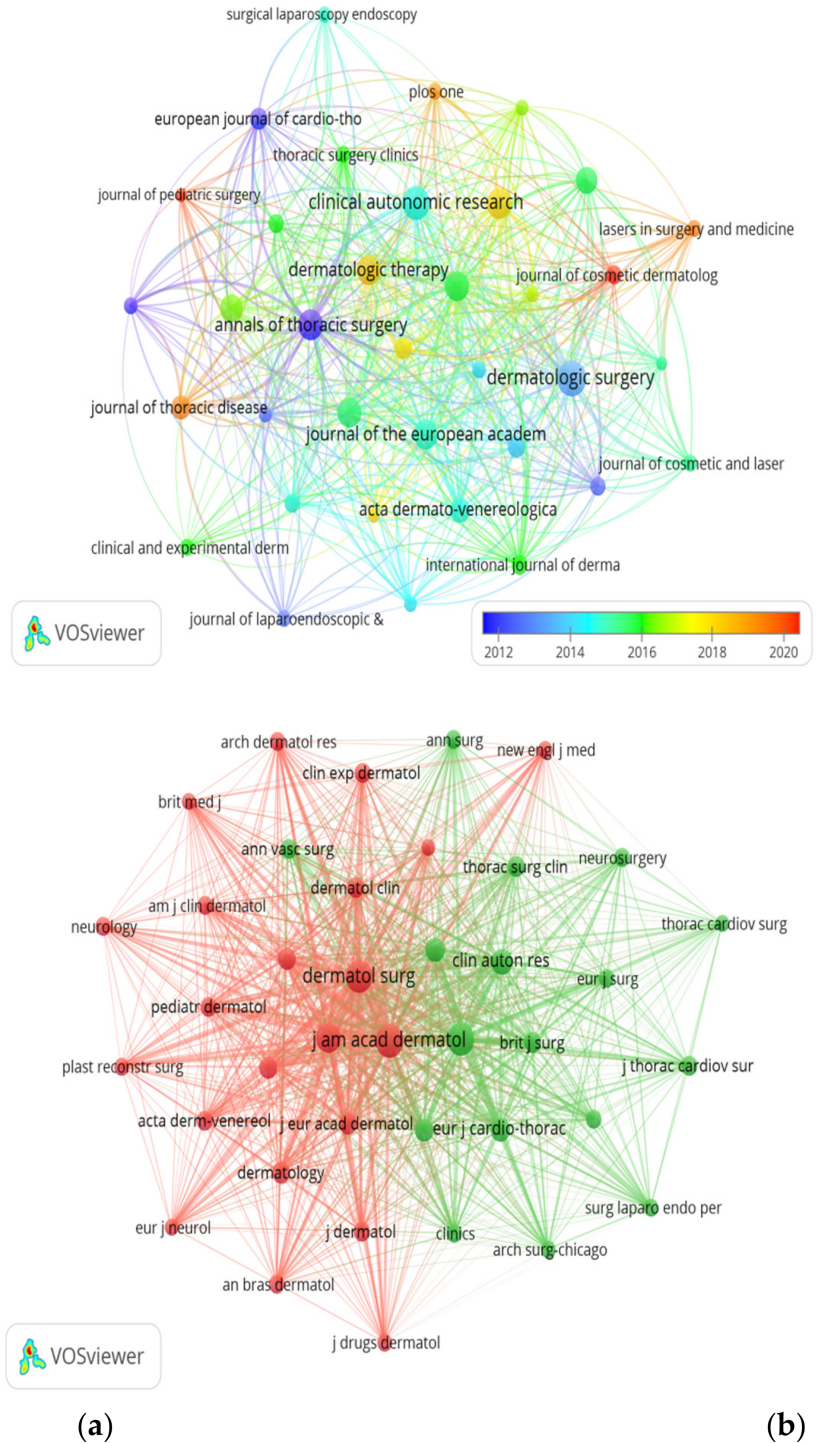
**(a)** Journals collaboration network visualization map. **(b)** Co-cited journals collaboration network visualization map.

After filtering out authors with less than 40 co-citations among the 5,128 co-cited authors, we identified 10 authors who were co-cited more than 145 times, as presented in Table 3. The author with the highest number of co-citations, 503, was Nelson Wolosker, followed by David R. Strutton with 250 co-citations, and José Ribas Milanez de Campos with 233 co-citations. Subsequently, we created a co-citation network, illustrated in Figure 5b, depicting active collaborations among various co-cited authors such as Clotilde Théry and Seon Hee Kim, and Bo Zhang and Ruenn Chai Lai. The network in Figure 6b underscores the interconnected web of collaborations within the academic community.

### 3.5 Journals and co-cited journals

Of the 226 academic journals that have published papers related to hyperhidrosis, the most prominent publications include Dermatologic Surgery, Clinical Autonomic Research, Annals of Thoracic Surgery, and Journal of Dermatology, with 25 (3.4%), 23 (3.2%), 20 (2.7%), and 20 (2.7%), respectively (Table 4). An analysis of the top 15 journals in terms of impact factors reveals that the Journal of the American Academy of Dermatology has the highest impact factor (IF = 12.8), followed closely by the Journal of the European Academy of Dermatology and Venereology (IF = 9.2). Subsequently, a screening process focused on 36 journals with a minimum of 6 relevant papers was conducted to map out the journal network (Figure 6a), revealing active citation relationships of Dermatologic Surgery with the Journal of the American Academy of Dermatology, Journal of Cosmetic and Laser Therapy, Clinics in Dermatology, and Journal of Dermatological Therapeutics. Regarding co-citations within the top 15 journals, four of them received more than 500 citations, with the Journal of the American Academy of Dermatology leading in the most citations (*n* = 1,235), followed by Annals of Thoracic Surgery (co-citation = 1,076), Dermatologic Surgery (*n* = 1,051), and the British Journal of Dermatology (*n* = 746). In addition to being highly esteemed in the field, the Journal of the American Academy of Dermatology boasts the highest impact factor (IF = 12.8), closely followed by the JAMA Dermatology (IF = 11.0). A co-citation network diagram (Figure 6b) was constructed by identifying journals with a minimum of 100 co-citations, revealing strong positive co-citation connections between the Journal of the American Academy of Dermatology and other influential publications such as Dermatologic Surgery, British Journal of Dermatology, and Annals of Thoracic Surgery.

**Table 4:** Distribution of the top 15 journals and co-cited journals on HH.

Rank	Journal	Count	IF	JCR	Co-cited journal	Co-citation	IF	JCR
1	Dermatologic Surgery	25	2.5	Q2	Journal of the American Academy of Dermatology	1,235	12.8	Q1
2	Clinical Autonomic Research	23	3.9	Q1	Annals of Thoracic Surgery	1,076	3.6	Q1
3	Annals of Thoracic Surgery	20	3.6	Q1	Dermatologic Surgery	1,051	2.5	Q2
4	Journal of Dermatology	20	2.9	Q2	British Journal of Dermatology	746	11.0	Q1
5	Journal of the American Academy of Dermatology	19	12.8	Q1	Clinical Autonomic Research	419	3.9	Q1
6	Journal of the European Academy of Dermatology and Venereology	19	9.2	Q1	European Journal of Cardio-Thoracic Surgery	349	3.1	Q1
7	Pediatric Dermatology	19	1.2	Q4	Surgical Endoscopy and Other Interventional Techniques	313	2.4	Q1
8	Dermatologic Therapy	19	3.7	Q1	Journal of Vascular Surgery	293	3.9	Q1
9	Thoracic and Cardiovascular Surgeon	17	1.3	Q3	Journal of the European Academy of Dermatology and Venereology	257	9.2	Q1
10	Journal of Drugs in Dermatology	16	1.5	Q4	JAMA Dermatology	257	11.5	Q1
11	Acta Dermato-Venereologica	14	3.5	Q1	International Journal of Dermatology	243	3.6	Q1
12	Journal of Thoracic Disease	13	2.1	Q4	Dermatology	236	3.0	Q2
13	Dermatologic Clinics	12	2.2	Q3	British Journal of Surgery	220	8.6	Q1
14	Journal of Dermatological Treatment	12	2.9	Q2	Thoracic Surgery Clinics	215	1.1	Q2
15	European Journal of Cardio-Thoracic Surgery	12	3.1	Q1	Dermatologic Clinics	213	2.2	Q3

### 3.6 Analysis of keywords

**Figure 7:**
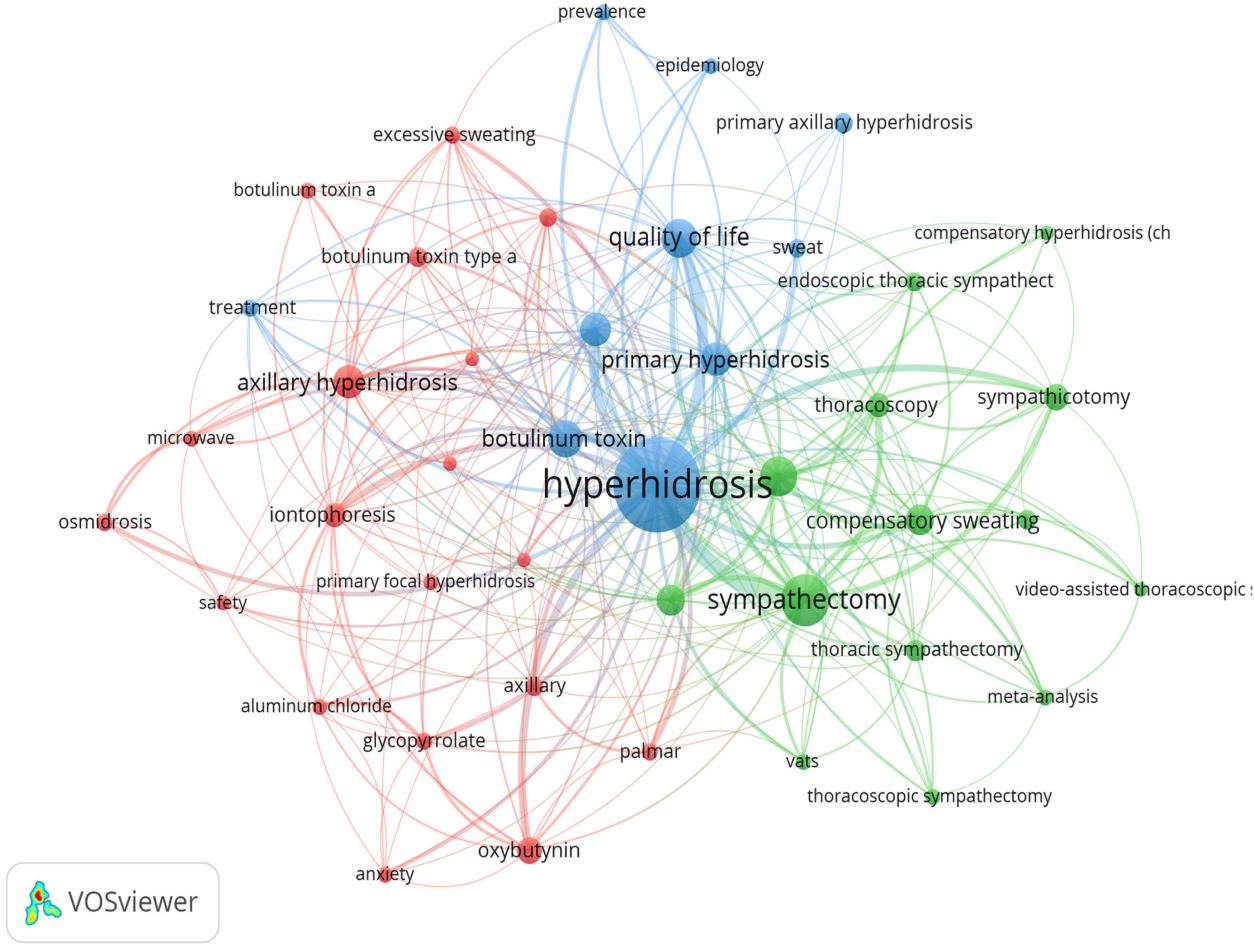
Co-occurrence network map of keywords.

**Figure 8:**
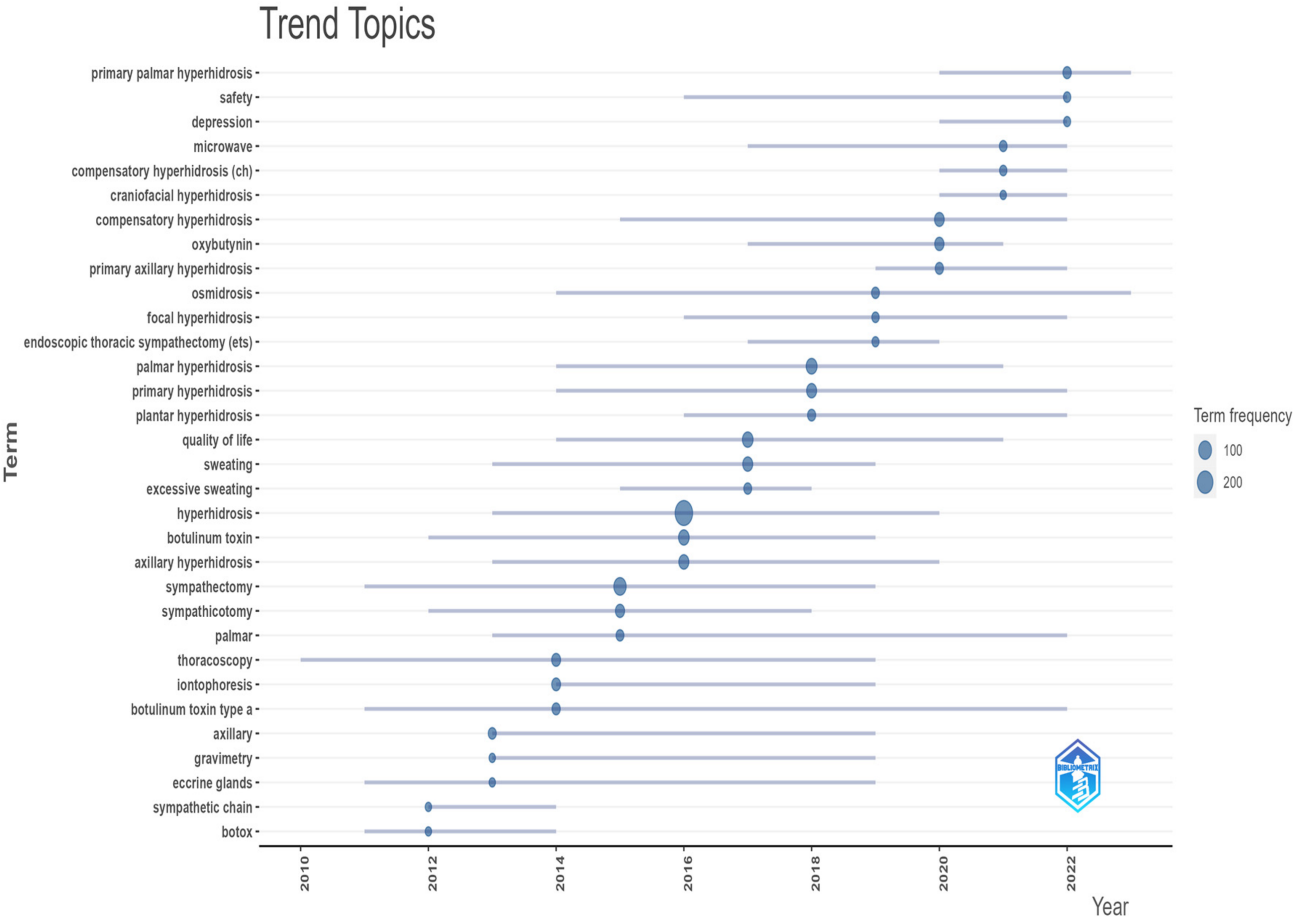
Trending topics of the keywords.

The top 20 high-frequency keywords in hyperhidrosis research, as displayed in Table 5, include sympathectomy, palmar hyperhidrosis, quality of life, and botulinum toxin, which appeared more than 45 times, indicating the primary research directions in hyperhidrosis. Utilizing VOS viewer, we conducted cluster analysis on keywords with seven or more occurrences, as shown in Figure 7. The visualization highlights three distinct clusters symbolizing different research directions. The green cluster encompasses keywords like sympathectomy, palmar hyperhidrosis, compensatory hyperhidrosis, endoscopic thoracic sympathectomy, and thoracoscopy. In contrast, the red cluster comprises keywords such as axillary hyperhidrosis, iontophoresis, oxybutynin, and plantar hyperhidrosis. Lastly, the blue cluster features keywords like quality of life, botulinum toxin, primary hyperhidrosis, and epidemiology. Analyzing the trending topics of the keywords between 2010 and 2019 (Figure 8), the research mainly centered around thoracoscopic surgery, with thoracoscopy and sympathectomy as key themes. However, a shift occurred post-2020, where scholars directed their focus towards postoperative complications and mental health in patients with hyperhidrosis. Key topics of interest include quality of life, safety, compensatory sweating, and depression, indicating the current research hotspots in hyperhidrosis.

**Table 5:** Top 20 keywords of HH from 2008 to 2023.

Rank	Keyword	Counts	Rank	Keyword	Counts
1	hyperhidrosis	288	11	oxybutynin	24
2	sympathectomy	85	12	sympathicotomy	23
3	palmar hyperhidrosis	52	13	thoracoscopy	20
4	quality of life	47	14	iontophoresis	19
5	botulinum toxin	46	15	thoracic sympathectomy	15
6	Axillary hyperhidrosis	36	16	primary palmar hyperhidrosis	14
7	sweating	35	17	botulinum toxin type a	13
8	primary hyperhidrosis	35	18	primary axillary hyperhidrosis	13
9	compensatory sweating	31	19	sweat	12
10	compensatory hyperhidrosis	30	20	plantar hyperhidrosis	12

## 4 Discussion

### 4.1 Basic information

To gain insight into the current state of research on hyperhidrosis, the study analyzed papers published between 2008 and 2023. The characteristics examined included year of publication, number of citations, country of origin, institution, journal, author, and subject. The ten most prolific publishers were from six different countries. The University of São Paulo stands out as the institution with the highest number of publications (*n* = 41) and the highest citation frequency (average citations: 26.51 per article) among the selected institutions. It is noteworthy that the Journal of the American Academy of Dermatology was identified as the primary venue for highly cited articles. Moreover, the study highlighted key researchers in the field, with Nelson Wolosker from the University of São Paulo leading the pack as the most published and cited author among those who contributed to the papers. He was closely followed by David R. Strutton from Saint Louis University. Co-cited references, fundamental to a field's research foundation, were also examined in this bibliometric study. A selection of the 10 most co-cited references provided insight into the underpinnings of hyperhidrosis research. The most cited publication in the hyperhidrosis domain was identified as Strutton DR's 2004 article titled “Prevalence of hyperhidrosis in the United States and the impact on patients with axillary hyperhidrosis: results of a national survey”. This study revealed that hyperhidrosis had a more significant impact on the US population than previously recognized, signaling a growing recognition of hyperhidrosis patients among scholars.

The United States has the most articles in hyperhidrosis research and has extensive collaborations with other countries/regions, indicating that the United States is a leader in this field. China has the second highest number of publications and has collaborated with several countries/regions, indicating that China has also made some achievements in hyperhidrosis research and is actively involved in international collaborations. With respect to academic institutions, the University of Pittsburgh has the highest number of publications; however, there is a paucity of collaboration between institutions. Regarding authors, collaboration is dispersed, with the absence of a substantial collaborative team. To promote the advancement of hyperhidrosis research, future efforts should focus on enhancing collaboration among institutions and authors, as well as on integrating available resources.

### 4.2 Hotspots and fronts

Based on our examination of co-cited literature, high-frequency keywords, and keyword clusters, the research focus in hyperhidrosis primarily centers on pharmacological treatment, surgical treatment, and postoperative complications. In each research hotspot, the most significant research items were botulinum toxin, sympathectomy, and compensatory sweating.

#### 4.2.1 Pharmacological treatment

Topical antiperspirants are often recommended as the initial treatment for mild hyperhidrosis due to their effectiveness in blocking distal sweat glands, leading to the degeneration of epithelial and secretory cells, thereby inhibiting local sweating (22, 23). A randomized controlled trial (RCT) investigated the impact of topical aluminum salts on axillary hyperhidrosis, treating 20 patients with aluminum chloride lotion 20% and aluminum sesquichlorohydrate lotion 20% daily in different axillae. Both treatments significantly reduced the Hyperhidrosis Disease Severity Scale (HDSS) from a mean of 3.5 points to 1.25 points after 2 weeks (24). Glycopyrronium bromide, a topical anticholinergic drug, functions by blocking acetylcholine action to inhibit local sweating. Studies have examined its efficacy and safety in two RCTs. The first RCT with 30 patients demonstrated that daily application of glycopyrronium bromide 1% or 2% reduced sweating by 75% in 87.5% of patients after 8 days, surpassing the placebo group results (25). The second RCT with 171 patients showed a significant reduction in axillary sweating levels with daily administration of 1% glycopyrronium bromide compared to the placebo group (26). Topical oxybutynin, another anticholinergic agent, competitively inhibits acetylcholine, thus reducing local sweating. In an RCT with 53 patients, the application of topical oxybutynin gel 10% twice daily led to a notable improvement in both the Hyperhidrosis Disease Severity Scale and the Dermatology Life Quality Index (27). Botulinum Toxin Type A (BTX type A) Liposomal Cream blocks acetylcholine release, effectively preventing local sweating. In a study involving 20 patients, the daily application of BTX type A liposomal cream proved more effective than placebo in reducing perspiration, as measured by Trans epidermal Water Loss (TEWL) at week 2 and week 8 (28). Furthermore, the efficacy of botulinum toxin in treating hyperhidrosis may be influenced by the dosage administered (29). Recent studies have demonstrated the efficacy of a combination of fractional laser therapy and botulinum toxin in the management of hyperhidrosis (30). Furthermore, the injection of botulinum toxin type A, facilitated by carbon dioxide fractional laser technology, has been shown to extend disease-free survival (31). Iontophoresis, on the other hand, involves exposing the skin to a weak electric current to alter the local pH, block sweat ducts, or inhibit the sympathetic nervous system (32). Sofpironium topical gel is a retrometabolically-designed topical anticholinergic with rapid metabolism, which is associated with reduced side effects and targeted efficacy. For the pooled co-primary endpoint of gravimetric sweat production at treatment end, the treatment group had greater reduction in sweat production (*p* = 0.0002). Secondary endpoints also showed a statistically significant benefit for sofpironium topical gel vs. control. Treatment was well-tolerated (33, 34). While new treatment options like microwave devices (35), diode lasers (36), and segmented microneedle radiofrequency (FMR) (37) have emerged to address hyperhidrosis, their efficacy and safety require further investigation through rigorous research to provide substantial evidence supporting their effectiveness.

#### 4.2.2 Surgical treatment

Surgical treatment is primarily recommended for hyperhidrosis patients who have not responded to non-surgical treatments or are not suitable for them (38). However, surgery should be considered a second or third-line treatment due to its invasiveness and risk compared to other treatments (39). The present study will compare the efficacy of fractional microneedle radiofrequency with that of botulinum toxin type A in the treatment of primary axillary hyperhidrosis. This condition has been observed to be more amenable to non-surgical treatment (40). The most used surgical methods for treating hyperhidrosis currently are Endoscopic Thoracic Sympathectomy (ETS), CT-guided sympathectomy with radiofrequency (RFS), and local suction (41–43). ETS is the standard surgical treatment for hyperhidrosis of the palms, face, and axillae, with a success rate of over 95% (44). The preferred surgery to treat palmoplantar hyperhidrosis is to excise the sympathoadrenal chain at the R4/R5 levels. In contrast, ETS is less effective in the treatment of axillary hyperhidrosis and has a higher regret rate. Liposuction, the initial surgical procedure for primary localized hyperhidrosis, has now been modified to primarily treat axillary hyperhidrosis. Heidemann et al. compared the use of the localized scraping technique with ETS in treating patients with simple axillary hyperhidrosis, concluding that the former produced better local results and fewer adverse effects (45). However, mild recurrence of symptoms is more common with localized scraping. Therefore, it is recommended to use localized scraping for patients with simple axillary hyperhidrosis and to reduce the use of ETS at the R2–3 or R2–4 levels. Minimally invasive surgery has advanced the treatment of hyperhidrosis in recent years. Radiofrequency ablation is reported as a new treatment option for patients with poor drug control, offering advantages such as less damage, faster recovery, lower cost, and precise efficacy. In a study of 370 patients (740 sides) undergoing CT-guided percutaneous radiofrequency sympathectomy (RFS), RFS was successfully performed on 637 sides, with a technical success rate of 86.1%, indicating that it is a safe and effective method for symptomatic relief in patients with hyperhidrosis (46). A number of studies have been conducted that compare pharmacologic treatment with surgical treatment. The prevailing treatments for axillary hyperhidrosis, characterized by the presence of small sweat gland orifice obstruction and small sweat gland duct atrophy, are Botulinum toxin A (BTX) and microwave thermolysis (MWT). A comparative analysis revealed that 75% of axillae treated with MWT exhibited these characteristics, compared to the 75% observed in BTX-treated axillae (47).

#### 4.2.3 Postoperative complication

Compensatory sweating (CS) is a prevalent issue post-sympathectomy for hyperhidrosis, potentially leading to decreased patient satisfaction. Compensatory sweating, a phenomenon observed in the aftermath of hyperhidrosis surgery, frequently manifests in the chest, abdomen, back, and thighs. The severity of CS varies, affecting up to 98% of patients, with no established standard treatment (48). Existing literature suggests several treatments, albeit with limited data and uncertain outcomes. Factors contributing to CS encompass the extent of sympathetic chain manipulation, degree of denervation, and body mass index. Some studies propose a correlation between patients' psychological traits and CS development (49, 50). Treatment options span non-surgical interventions, such as topical medications, botulinum toxin, systemic anticholinergic drugs, and iontophoresis, alongside surgical interventions like splenectomy and extended sympathectomy. However, empirical support for their efficacy remains inadequate. Han et al. introduced an innovative sympathectomy method targeting R8 or even R12 to mitigate severe CS, exhibiting noteworthy reductions in CS severity with no severe cases or major complications reported (51).

### 4.3 An examination of the extant research on hyperhidrosis

Recent therapeutic developments for hyperhidrosis encompass a variety of approaches, including gene therapy (currently in the research phase but showing considerable potential), novel anticholinergics (e.g., sofpironium bromide gel, approved for specific patient populations), fractional microneedle radiofrequency (FMRF), radiofrequency ablation (RFAB), and CT-guided radiofrequency thermocoagulation (CT-thermo-coagulation). These methods have demonstrated a reduction in trauma, accelerated recovery, and enhanced effectiveness (1). The broad potential for interdisciplinary applications is evident. For instance, the convergence of dermatology and genetics facilitates a more nuanched understanding of pathogenesis, thereby allowing for customized treatment approaches. Similarly, the collaboration between molecular biology and clinical medicine fosters the development of novel therapeutic agents and diagnostic techniques. Additionally, the integration of psychology and medicine leads to comprehensive treatment strategies that address both psychological and physiological dimensions of health (2). In the domain of policy evolution, there will be an ongoing refinement of drug regulatory policies, leading to enhanced approval processes and standards. Additionally, there will be a regulation of the use of emerging minimally invasive technologies under medical technology access policies. Furthermore, health insurance policies are likely to undergo adjustments in response to the development of therapeutic approaches and various other factors. The effective incorporation of tools in reimbursement will serve to enhance patient access.

As medical technology continues to advance, such as with gene technology and nanotechnology, new opportunities for research on hyperhidrosis may emerge. For instance, through genetic research, we can gain a more profound understanding of the pathogenesis of hyperhidrosis, which will provide a theoretical foundation for the development of novel treatments. Moreover, by leveraging nanotechnology, we can engineer a novel drug delivery system to enhance the efficacy of pharmaceutical therapy. Moreover, the future of hyperhidrosis research will be marked by an emphasis on multidisciplinary collaboration and the integration of knowledge from multiple fields, including medicine, biology, and psychology, to provide a comprehensive understanding of the etiology, pathogenesis, treatment, and prevention of hyperhidrosis.

## 5 Conclusions

Currently, the leading countries in terms of the number of articles, researchers, and institutions are the United States and China. Dermatologic Surgery is projected to have the largest number of published papers from 2008 to 2023. The most common academic focus within this field continues to be the treatment of hyperhidrosis and the management of compensatory hyperhidrosis. Despite this academic dominance, there is still a pressing need to enhance collaboration and exchange between different countries and institutions.
